# Built Shallow to Maintain Homeostasis and Persistent Infection: Insight into the Transcriptional Regulatory Network of the Gastric Human Pathogen *Helicobacter pylori*

**DOI:** 10.1371/journal.ppat.1000938

**Published:** 2010-06-10

**Authors:** Alberto Danielli, Gabriele Amore, Vincenzo Scarlato

**Affiliations:** 1 Department of Biology, University of Bologna, Bologna, Italy; 2 Animal Physiology and Evolution Laboratory, Stazione Zoologica Anton Dohrn, Napoli, Italy; University of California San Diego, United States of America

## Abstract

Transcriptional regulatory networks (TRNs) transduce environmental signals into coordinated output expression of the genome. Accordingly, they are central for the adaptation of bacteria to their living environments and in host–pathogen interactions. Few attempts have been made to describe a TRN for a human pathogen, because even in model organisms, such as *Escherichia coli*, the analysis is hindered by the large number of transcription factors involved. In light of the paucity of regulators, the gastric human pathogen *Helicobacter pylori* represents a very appealing system for understanding how bacterial TRNs are wired up to support infection in the host. Herein, we review and analyze the available molecular and “-omic” data in a coherent ensemble, including protein–DNA and protein–protein interactions relevant for transcriptional control of pathogenic responses. The analysis covers ∼80% of the annotated *H. pylori* regulators, and provides to our knowledge the first in-depth description of a TRN for an important pathogen. The emerging picture indicates a shallow TRN, made of four main modules (origons) that process the physiological responses needed to colonize the gastric niche. Specific network motifs confer distinct transcriptional response dynamics to the TRN, while long regulatory cascades are absent. Rather than having a plethora of specialized regulators, the TRN of *H. pylori* appears to transduce separate environmental inputs by using different combinations of a small set of regulators.

## Introduction

*Helicobacter pylori* is a Gram-negative bacterium, recognized as the principal causative agent of chronic inflammation of the mucous membranes of the human stomach and of peptic ulcer worldwide [1], [2]. It is one of the major risk factors for gastric neoplasia, including lymphomas and gastric cancers [3]. Survival in the gastric niche depends on the concerted expression of virulence factors and housekeeping genes, which enables *H. pylori* to withstand the stresses imposed by the harsh acidic environment and to counteract the host's responses [4], [5]. The restricted gastric habitat of *H. pylori* has been associated with reduced functional redundancy of its small genome (∼1.6 Mb), characterized by a constrained number of transcriptional regulators [6], [7]. In analogy to other bacteria, the environmental signals are transduced into coordinated output expression by a transcriptional regulatory network (TRN) that can be reconstructed by describing how transcriptional regulators and target genes are interconnected in local network structures [8]–[10].

**Figure 1:**
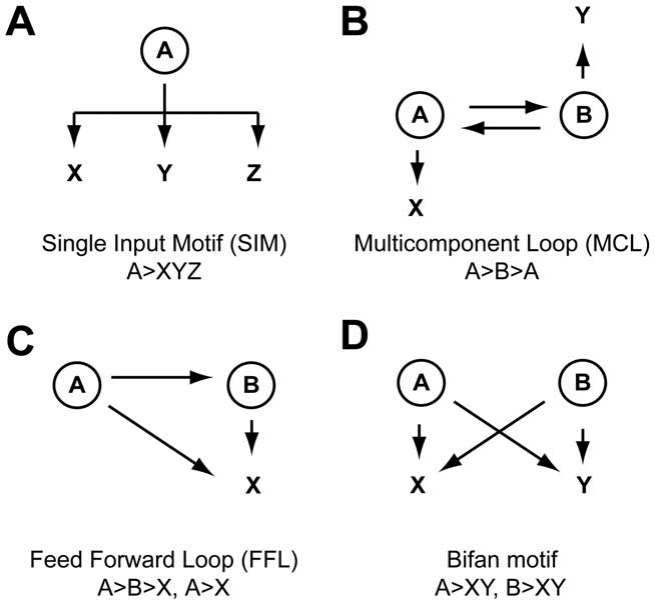
Networks motifs found in the *H. pylori* TRN. **A**. Single input motif (SIM); a single transcription factor A regulates a set of operons (X, Y, Z); frequently A is autoregulated. **B**. multicomponent loop (MCL); transcription factor A regulates another regulator B, which in turn can regulate the transcription of A; each regulator can also control transcription of a separate set of target operons. **C**. Feed forward loops (FFLs) are three node motifs, occurring with high frequency in prokaryotic TRNs. A top regulator, A, which controls a second regulator, B, and a target gene, X, which is regulated by both A and B. The regulators in this motif (A, B) have an asymmetric position and hierarchy, as A regulates two targets, while B only one. According to the net signs feeding into X, FFL can be coherent (same sign) or incoherent (opposite sign), negative or positive, conferring different response kinetics [13] **D**. Bifan motif (BM); a set of operons (X, Y) is each regulated by the combination of two (A, B; BM) or multiple (A, B, C, etc.; multi input motif, not shown) transcription factors.

TRNs commonly exhibit multilayered hierarchical structures, composed of regulatory modules (origons) that include the concerted activity of transcription factors (TFs) regulating related physiological functions [11]. Origons therefore embody regulatory sub-networks that originate at a distinct class of sensor TFs, rooting a signal input node [12]. Each origon comprises a master regulator, sensing a specific signal, followed by intermediate TFs and regulatory interactions that ultimately feed into output gene targets. Within or between origons, TFs and target genes are wired in specific local patterns of interconnection termed motifs (Figure 1). They enable the system to respond with specific dynamics, according to the biological nature of the triggering signal [13]. Interestingly, it has been shown that extensive tinkering of transcriptional interactions at the local level has contributed to the evolution of prokaryotic TRNs by wiring orthologous genes in different types of motifs [14]. In fact, these local network structures do not evolve as rigid units with fixed patterns, but tend to be conserved in organisms sharing similar living environments, regardless of their phylogenetic distance [15]. Accordingly, identification of definite motifs and TRN organization in *H. pylori* may represent important findings for understanding the regulatory circuits of other human microbial pathogens.

## The *H. pylori* TRN

For the analysis, we have modeled the *H. pylori* TRN using BioTapestry software [16], by integrating the regulatory connections gathered from molecular, transcriptome, and ChIP-chip data available in the literature. To filter out indirect connections and pleiotropic effects, regulatory interactions were considered only for targets subjected to direct transcriptional control, defined by ascertained TF binding to regulatory elements of the target gene. We examined 685 non-redundant transcriptional regulations and identified 224 direct interactions (Dataset S1), separated according to the sensor TF serving as rooting node of the origon. The model, supplied as an interactive Java plug-in file (Model S1, Text S1), covers approximately 80% of the annotated regulators in *H. pylori*, a value that exceeds that reported for the most studied model organism *Escherichia coli* (50% coverage) [11], where hundreds of regulators make the dissection of the TRN difficult.

In fact, *H. pylori* appears to enroll only 17 bona fide TFs (Table 1), encompassed in four main origons, which process the key physiological responses needed to colonize the gastric niche: 1) heat and stress response, 2) motility and chemotaxis, 3) acid acclimation, and 4) metal ion homeostasis.

**Table 1:** *H. pylori* transcription factors and regulators.

TF	Gene	Description	Function	Origon	Motif	Target TFs
**σ^80^**	*rpoD (HP0088)*	Vegetative sigma factor	Housekeeping	-	-	All; except *fliA ?* (upstream RpoN consensus)
**σ^54^**	*rpoN (HP0714)*	Alternative sigma factor	Flagellar regulation; *class II* genes; basal body, hook	FLAG	SIM	*fliA*; based on upstream RpoN consensus sequence
**σ^28^**	*fliA (HP1032)*	Alternative sigma factor	Flagellar regulation; *class III* genes; late structures	FLAG	SIM	
**FlgR**	*flgR (HP0703)*	NtrC-like response regulator	Flagellar regulation; RpoN-dependent regulation	FLAG	SIM	*fliA*; based on upstream RpoN consensus sequence
**FlgS**	*flgS (atoS) (HP0244)*	NtrB-like cytoplasmic histidine kinase	Flagellar regulation; acid acclimation	FLAG	P∼	P∼FlgR; P∼ArsR (based on genetic evidence)
**CheA**	*cheA (HP0392)*	Histidine kinase	Chemotaxis	FLAG		P∼CheY; P∼CheY2
**CheY**	*cheY (HP1067)*	Response regulator	Chemotaxis	FLAG	P∼	-
**CheY2**	*(HP0392)*	CheY-like reciever domain fused to CheA	Chemotaxis; P∼sink	FLAG	P∼	-
**ArsR**	*arsR (HP0166)*	ompR-like response regulator	Acid acclimation	pH	SIM	*arsR*; essential gene
**ArsS**	*arsS (HP0165)*	Transmembrane histidine kinase	Acid acclimation; periplasmic acid sensor	pH	P∼	P∼ArsR
**HP1021**	*(HP1021)*	Atypical orphan response regulator	Acetone metabolism	NA	NA	NA; essential gene
**HP1043**	*(HP1043)*	Atypical orphan response regulator	Growth	NA	NA	*HP1043*; essential gene
**CrdR**	*crdR (HP1364)*	Response regulator	Copper resistance	NA	NA	NA
**CrdS**	*crdS (HP1365)*	Histidine kinase	Copper resistance	NA	P∼	P∼CrdR
**Fur**	*Fur (HP1027)*	Ferric uptake regulator	Pleiotropic; metal ion homeostasis	METAL	BM; MCL; FFL	*fur*, *nikR*, *arsR*, *rpoN* a, *flgS* a, *hrcA* a
**NikR**	*nikR (HP1338)*	*E. coli* NikR homologue	Pleiotropic; nickel ion homeostasis	METAL	BM; MCL; FFL	*nikR*, *fur*
**HrcA**	*hrcA (HP0111)*	*B. subtilis hrcA* repressor homologue; membrane associated	Heat shock; stress response	HS	FFL	*hrcA*
**HspR**	*hspR (HP1025)*	*Streptomyces* spp. *hspR* repressor homologue	Heat shock; stress response	HS	FFL	*hspR*, *hrcA*

a Evidence gathered from ChIP-chip experiments. Note that many transcription factors, especially repressors, are autoregulated, and that Fur and NikR are wired in a multicomponent feedback loop (MCL), which is rarely found in bacteria.

BM, bifan input motif; FFL, feed forward loop; FLAG, flagellar biosynthesis and motility origon; HS, heat shock and stress response origon; MCL, multicomponent loop; METAL, metal-ion homeostasis origon; motif, type of local network structure or interconnnection in the main origon; P∼, phospho-transfer activation; pH, acid acclimation origon; SIM, single input motif; TF, transcription factor.

### The Heat Shock and Stress Response Origon

**Figure 2:**
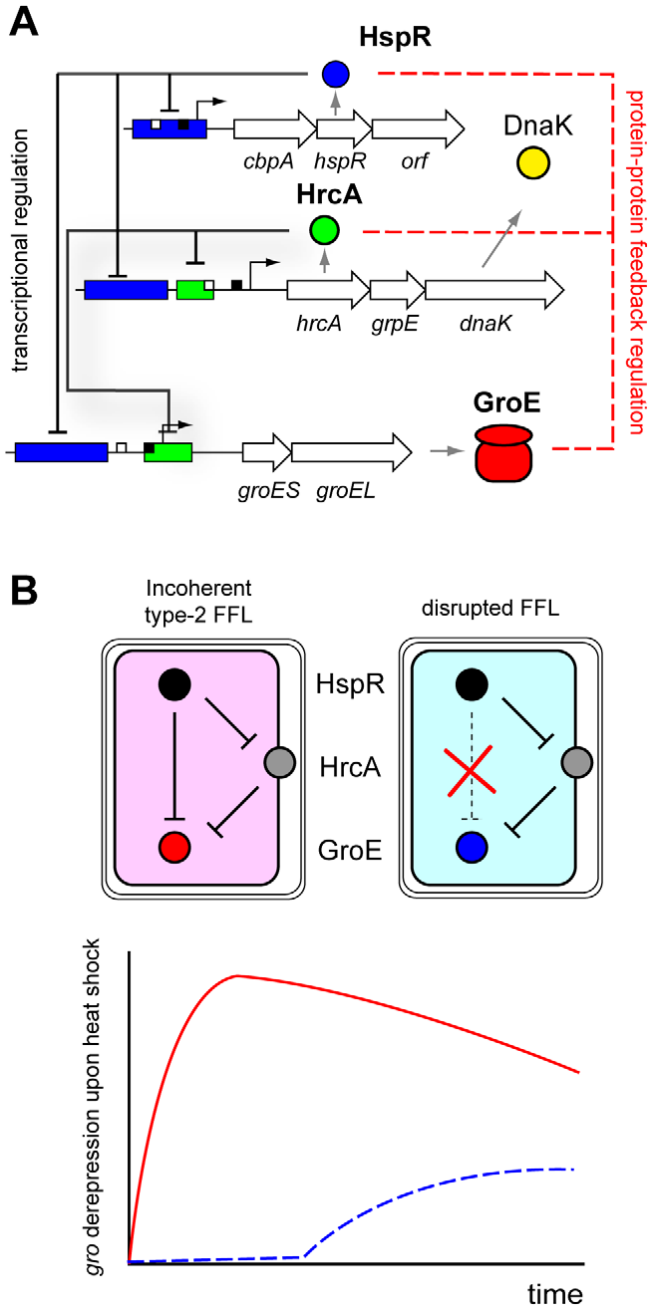
The heat shock and stress response origon. **A**. The heat shock origon is composed of two TFs (HspR and HrcA) repressing directly three main target operons: I) the *cbp* operon, encoding, respectively, a DnaJ-homologue CbpA, the HspR regulator itself, and a gene product of unknown function with homology to a helicase (HP1026); II) the *hrcA* operon, coding for the HrcA regulator, DnaK, and the GrpE co-chaperone; and III) the *groESL* operon, encoding conserved GroES and GroEL chaperones. Block arrows: open reading frames. Green boxes: HrcA operators; blue boxes, HspR operators. −10 and −35 boxes are depicted by black and white squares, respectively. TF–DNA interactions and direct transcriptional control are depicted by black lines; red dotted lines represent protein–protein interactions important for feedback control of the circuit; arrowheads denote positive regulation; hammerheads indicate negative regulation. **B**. Incoherent type-2 FFL wiring of HspR, HrcA, and the *groESL* operon, responsible for the prompt derepression kinetics of P*_gro_* upon heat shock. Red line: derepression kinetic of the intact FFL motif; blue dotted line: derepression kinetic of a mutated P*_gro_* promoter in which the HspR operator has been deleted. The membrane association of HrcA in *H. pylori* [47] may confer additional sensory potential to the circuit, e.g., through sensing of periplasmic misfolded peptides, or need to release HrcA from the inner membrane by a stress-inducible co-factor.

The heat shock origon is amongst the best-understood regulatory modules in *H. pylori*. Whereas most Gram-negatives employ specialized sigma factors (σ^32^) to positively regulate the transcriptional responses to heat shock, *H. pylori* has evolved an opposite strategy, commonly found in Gram-positive bacteria, that implements two repressors with homology to *Bacillus subtilis* HrcA [17], [18] and *Streptomyces* spp. HspR [19]. Specifically, the heat shock origon of *H. pylori* is composed of two TFs (HspR and HrcA) directly repressing three main target operons, including the *groESL* chaperone genes (Figure 2A). All three operons are responsive to heat shock and are activated by the presence of misfolded proteins or stress signals [20], [21]. HspR alone represses transcription of the *cbpA* operon, thereby negatively autoregulating its own synthesis [22]. On the contrary, both HspR and HrcA are required for dual repression of the *groESL* and *hrcA* operons [23]. The DNA-binding activity of both repressors is enhanced by the product of the *groESL* target gene [24], suggesting that HrcA and HspR are involved with the GroE chaperonin system in a feedback regulatory loop, complying with the *B. subtilis* “titration model” [25]. This model postulates that upon heat shock, GroESL is titrated away by misfolded polypeptides, thereby relieving HspR/HrcA repression, and triggering the stress response.

Interestingly, although both TFs are required for full regulation, binding in vitro to their respective adjacent operators occurs in an independent, non-cooperative manner. A coherent explanation for two biochemically independent but functionally interconnected regulators can be evinced by the logical scheme of the heat shock response circuit (Figure 2B). In fact, the heat shock module is wired in what appears to be an exquisite example of an incoherent feed forward loop (FFL, Figure 1C). Incoherent FFLs greatly accelerate the response kinetics of a regulation cascade at the same steady state levels [13], [26]. Accordingly, in wild-type *H. pylori* strains the P*_gro_* promoter is promptly derepressed (<120 seconds) upon temperature shift to 42°C [20]. In a mutant in which the binding site of HspR has been deleted, and HrcA is still functional, the derepression of the P*_gro_* promoter is observed only after 60 minutes of temperature up-shift [23]. These data strongly argue in favor of a bona fide incoherent type-2 FFL motif governing GroESL chaperone synthesis. This has important implications, as the altered homeostasis of chaperones appears to indirectly influence virulence gene expression, as well as the assembly/expression of the flagellar apparatus (see below). Interestingly, the incoherent type-2 FFL is absent in *E. coli* (0 out of 42 described FFLs) and rare in *Saccharomyces cerevisiae* (3/56) [26]. Thus, the HspR/HrcA FFL may reflect evolutionary adaptation to the host environment that *H. pylori* encounters as a pathogen. In this light, it is interesting to recall that a similar heat shock regulation mode has been reported in *Staphylococcus aureus* (CstR/HrcA) [27].

### The Flagellar Biosynthesis Module

Flagellar, chemotaxis, and motor protein encoding genes are key virulence factors in *H. pylori*. Their deletion results in attenuated infections in animal models [28]–[30], possibly because of a failure to move in response to favorable or noxious gradients. In contrast to other well-characterized systems, the ∼40 motility genes of *H. pylori* are unclustered, frequently scattered within multicistronic gene operons [6], [31], [32]. Along with other pleiotropic regulatory effects, such as polarity and DNA supercoiling [33], their transcription is hierarchically regulated, employing a remarkable fraction of dedicated TFs (7 out of 17, Table 1).

As for other bacteria, flagellar genes are typically positively regulated and hierarchically organized in three main classes according to their activating sigma factor [32], [34]: *class I* encompasses gene targets transcribed by the vegetative σ^80^-containing RNA polymerase (RNAP), and comprises mostly flagellar regulatory genes (*flgR*, *flgS*, *rpoN*, *flhA*); *class II* includes specific targets of the alternative σ^54^ factor (RpoN), and encode components of the flagellar basal body and hook; finally, *class III* genes encode late flagellar structures, transcribed by σ^28^-(FliA)-containing RNAP. Other regulators include the FlgRS two-component system constituted by a NtrB-like cytoplasmic sensory histidine kinase (FlgS) and a NtrC-like response regulator (FlgR) [35]–[37], and the CheA/CheY/CheY2 system regulating chemotactic responses [29], [38].

**Figure 3:**
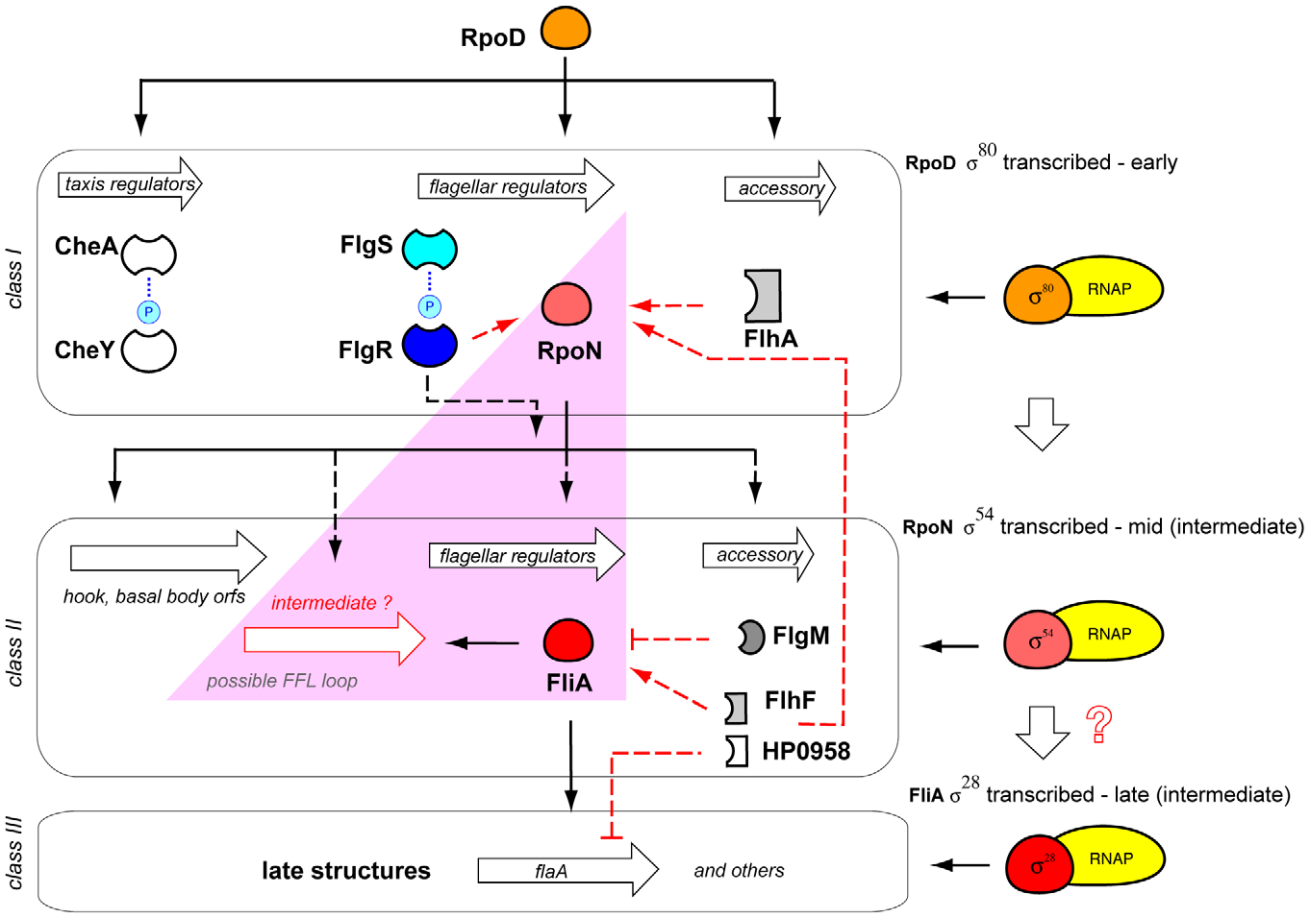
The flagellar biosynthesis module. RNAP, RNA polymerase; phosphorylation events are indicated by a light blue circle. CheAY, two component system involved in chemotaxis; FlgS, NtrB-like cytoplasmic histidine kinase; FlgR, NtrC-like response regulator. FlgM, anti-σ^28^ factor. FlhA, FlhF, HP0958: accessory factors. Note sequential activation of *class I*, *II*, and *III* due to σ regulatory cascade. A pink triangle depicts a putative coherent FFL with OR logic proposed to control expression of *intermediate class* flagellar genes [32].

Notably, a master regulator similar to the enterobacterial FlhDC is absent, possibly because motility and chemotaxis are essential in vivo and need to be constitutively expressed in *H. pylori*. Conversely, the flagellar regulatory module adopts a short σ regulatory cascade (σ^80^>σ^54^>σ^28^) initiated by the housekeeping σ^80^ factor (Figure 3), where each σ factor activates a dedicated target gene class with single input motif (SIM) circuitry (Figure 1A). SIMs frequently occur in systems expressing gene products that form assemblies with controlled stoichiometry [39], and exhibit time-shifted dynamics [40], [41]. Thus, the flagellar regulatory chain of *H. pylori* is appropriate to guarantee the correct sequential expression of early, middle, and late flagellar components. In analogy to *E. coli* [42], a putative type-1 coherent FFL with OR logic found at the bottom of the σ cascade may contribute to regulate an *intermediate class* of flagellar genes [32], and to sustain flagellar gene expression in the presence of discontinuous triggering signals. However, formal demonstration of the dynamic responses governed by this FFL has yet to be provided. In fact, despite the presence of a σ^54^ promoter consensus sequence upstream of the *fliA* gene, a direct regulation of RpoN on *fliA* is elusive to date [32], [43].

Besides TF–promoter (protein–DNA) interactions, several accessory proteins involved in post-transcriptional feedback mechanisms have been described: the anti-σ^28^ factor FlgM, itself transcribed as a *class II* gene, is involved in FliA inhibition of *class III* gene transcription [44], [45]. In addition, components of the basal body, FlhA and FlhF, appear to modulate *rpoN*- and *fliA*-dependent transcription of middle and late flagellar genes [32]. Moreover, the HP0958 protein described as an RpoN chaperone [46] also acts as an mRNA decay-promoting factor for the *flaA* transcript [43]. Thus, although the exact molecular mechanisms are not fully understood, it is clear that, in analogy to the heat shock origon controlled by GroESL levels, protein–protein interactions of accessory factors with TFs are central for feedback modulation of the flagellar regulatory circuit. Ectopic regulatory inputs, deriving from external origons, also feed into the flagellar circuit (see below). In addition, the output of the HspR-HrcA module indirectly affects motility, likely through altered flagellar biosynthesis occurring in strains where the intracellular levels of chaperones are deregulated [47]. This links the flagellar circuit with the sensing of stresses.

### The Acid Acclimation Origon

The capacity to grow under the harsh acidic conditions encountered in the stomach is a distinctive feature of *H. pylori* and is associated with virulence [48], [49]. Accordingly, the regulated expression of a dedicated set of so-called acid acclimation genes (the *ure* urease operon, aliphatic amidases *amiE* and *amiF*, arginase *roc*, etc.) allows *H. pylori* to keep acidity of the bacterial periplasm close to neutrality, and to maintain physiologic pH levels in the cytoplasm in the presence of urea and urease activity [50]–[52]. Transcription of acid acclimation operons is under control of the housekeeping σ^80^ factor and regulated principally by the essential acid response regulator ArsR [53]. ArsR is autoregulated and is encoded by an operon that also encompasses the cognate transmembrane ArsS histidine kinase [54]. It has been proposed that the signal sensed by ArsS is acidification of the periplasm, transduced through changes in protonation of several histidine residues (pKa ∼6.0) encompassed in the extracytosolic sensory domain [55]. This stimulus triggers phosphorylation of ArsR, thereby promoting its DNA-binding activity towards a specific set of promoters [56], [57]. However, whilst *arsR* appears to be an essential gene, strains carrying a point mutation that disrupts the phosphoacceptor site in the ArsR receiver domain, as well as *arsS* deletion mutants, are viable [57], [58]. Despite some discrepancies in the experimental datasets of several systematic studies, possibly arising from the use of diverse strains [53], [55]–[57], [59], [60], the emerging evidence points to the existence of distinct regulatory targets, which are controlled according to the phosphorylation status of ArsR:

- A first cluster of genes encompasses P∼ArsR-dependent target operons regulated by ArsR in a phosphorylation-dependent manner, upon mild acidification of the periplasm through ArsS signalling (*omp11*, *carbonic anhydrase*, *hypA*, *ureAB*).
- A second cluster of genes contains target operons that are regulated by more harsh acidic conditions, promoting acidification of the cytoplasm. Their regulation is P∼ArsR-dependent and phosphorylation of the regulator similarly promotes high affinity DNA binding to their promoters. However, they are not deregulated in *arsS* deletion mutants, and may therefore rely on a different (cytoplasmic) acid signal transducer to promote the phosphotransfer needed to activate ArsR. This group includes other genes central to the acid acclimation process such as *amiE*, *amiF*, and others.
- Finally, a third group of genes includes targets of unphosphorylated ArsR (including the *arsRS* operon) and whose regulation is not necessarily pH dependent. The latter group contains genes of unknown function that appear to be essential for viability.

Although a unifying consensus sequence has not been defined yet, it appears that the DNA elements recognized by either unphosphorylated or phosphorylated ArsR may be characterized by distinctive features, such as extension of the footprint and nucleotide sequence [53]–[55]. This substantiates the hypothesis of a bipartite ArsR regulon, which controls through distinct SIMs transcription of different sets of genes, according to the phosphorylation status of the response regulator (Figure S1). Additional complexity is provided by the position of the binding site in the target promoter, according to which ArsR can act as inducer or repressor of transcription.

Very interestingly, a recent work identified FlgS, the aforementioned cytosolic NtrB-like histidine kinase belonging to the flagellar biosynthesis module, as being also essential for survival of *H. pylori* at low pH [61]. Although it is not known whether FlgS is able to trigger ArsR phosphorylation upon acidification of the cytoplasm, it may represent a good candidate as a cytosolic acid sensor feeding into the ArsR regulon.

### The Metal Homeostasis Origon

In many bacterial pathogens, including *H. pylori*, metal starvation triggers the expression of virulence factors, which enables them to compete with the host for these essential nutrients. On the other hand, metal ions are toxic if present intracellularly in high amounts. Therefore, their homeostasis must be tightly controlled [62]. Three systems are dedicated to this fundamental task in *H. pylori*: the CrdRS two-component system, the ferric uptake regulator (Fur) involved in iron homeostasis [63], and a homolog of the Ni-responsive NikR regulator of *E. coli* [64]. Whilst the only identified genes targets of the CrdRS system appear to be involved in copper resistance [65], Fur and NikR have been described as pleiotropic regulators.

Fur regulates genes involved in both Fe^2+^ uptake [66]–[68] and detoxification [69], [70]. Distinctively, the metal ion cofactor can act as co-repressor (holoFur repressed genes) or as inducer (apoFur repressed genes) [71], [72]. Thus, the same information (presence or absence of Fe^2+^ in the cell) translates into two opposite transcriptional outputs. In accordance with its pleiotropic role, exemplified by the observed competitive colonization defects of *fur* mutants [73], Fur is an abundant protein and binds to ∼200 target loci in vivo, including genes coding for other regulators (*rpoN*, *flgR*, *flgS*, *cheA*, *nikR*) [74]. Consequently, hundreds of genes deregulated by *fur* deletion have been reported, although not all appear to be direct targets of the regulator [74], [75]. This suggests that Fur has a very central role in the *H. pylori* TRN.

**Figure 4:**
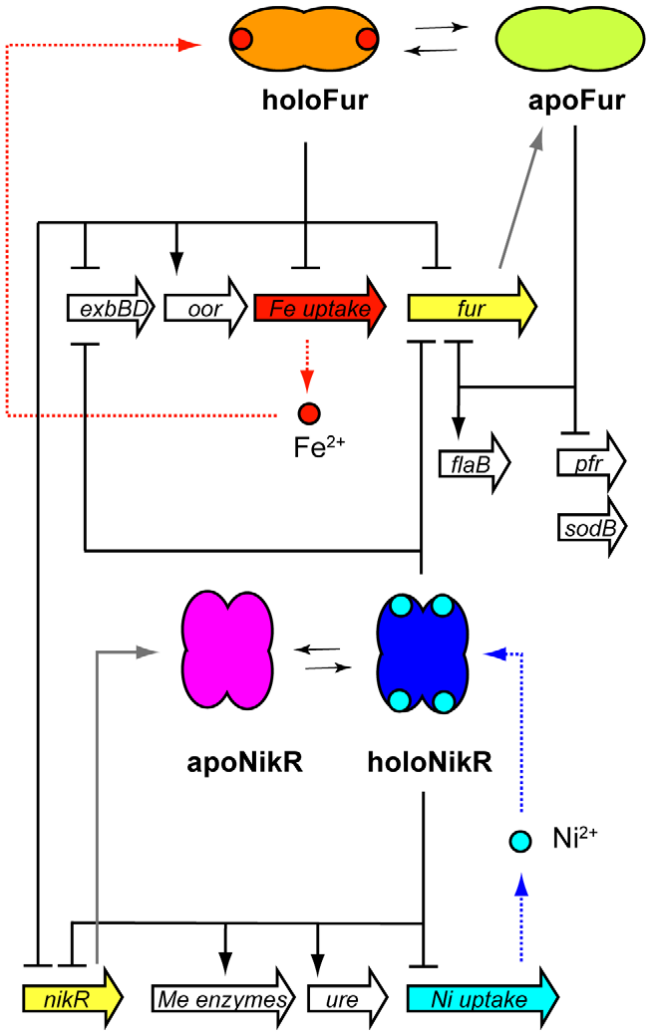
The metal homeostasis origon. Fur regulates transcription of metal transporters and siderophores (e.g., *frpB* and *fecA* paralogues), which need to be repressed upon iron repletion [66]–[68], as well as detoxifying genes that need to be promptly derepressed under the same Fe^2+^ replete conditions (e.g., *pfr* bacterioferritin and *sod* superoxide dismutase) [69], [70]. Fur generally acts as repressor, but it may also act positively on transcription on certain genes (*flaB*, *oor*) [74]. Similarly, NikR can act as positive or negative regulator of transcription, according to the position of the operator elements [79], [80]. With respect to the promoter, binding upstream appears to induce transcription of the *ureAB* operon in cultures grown in Ni^2+^ replete cultures [64]. Conversely, binding within the core promoter represses transcription, as shown for the *fecA3* and *frpB4* genes, encoding outer membrane proteins [68], [103], [105], the *nixA* permease gene [106], and the *exbBD-tonB* operon, that is involved in energization of outer membrane transport complexes involved in metal uptake [107], and itself part of the Fur regulon [74], [75], [81], [104]. Note that both Fur and NikR are autoregulated, and that they control reciprocally their transcription levels, through direct binding to their respective promoters [81]. Several operons such as *exbBD-tonB* are under the control of both Fur and NikR.

On the other hand, NikR mediates regulation of Ni^2+^ homeostasis in the cell, central to the activity of the nickel-enzyme urease. In contrast to Fur, apoNikR is unable to bind DNA, and Ni^2+^ coordination at high-affinity metal binding sites drives allosteric changes promoting the DNA binding activity of holoNikR [76]–[78]. According to the position of the operator elements, NikR can act as a positive or negative regulator of transcription [79], [80] (see also legend to Figure 4).

Together, Fur and NikR operate a bifan motif (BM; Figure 1D), which connects the metal responses in a horizontal and symmetric coordination logic with no obvious hierarchy (Figure 4). These motifs occur when multiple responses need to be coordinated to answer to mutual stimuli [40], as expected for metal ions, which play a key role in many different physiological processes. The metal homeostasis regulatory logic is further complicated by a multicomponent loop (MCL; Figure 1B), which directs reciprocal regulation of Fur and NikR [81]. In this scenario, regulation of shared targets can switch to a FFL motif as soon as the intracellular concentrations of metals reach threshold levels to cofactor the regulator DNA binding activity of either Fur or NikR. This provides a great flexibility to the circuit, especially in virtue of the capacity of Fur to bind specific promoters also in the apo-form [71].

Notably, regulatory connections emanate from the metal ion homeostasis module towards TFs and target genes encompassed in all other origons, making it a central “core” processor of the *H. pylori* TRN. Accordingly, *fur* deletion mutants are impaired in their acid tolerance [82], [83], and Fur in vivo targeting of ArsR has been reported in ChIP-chip experiments [74], substantiating a direct (and hierarchically important) role of Fur in the regulation of acid acclimation. The same study also revealed that protein levels of Fur increase in stationary phase, suggesting that this regulator is involved in the reported growth phase–dependent regulation of genes encoding metallo-proteins and iron-trafficking factors [68], [84], [85]. Similarly to Fur, NikR might be also involved in the regulation of several acid acclimation genes, including the urease operon [64], [86], possibly through increased bioavailability of the Ni^2+^ ion under low pH conditions, or through pH-responsive DNA binding activity, which has recently been reported [87]. In addition, the heat shock response origon is linked to the metal homeostasis circuit, as the heat shock operons are deregulated in *nikR* deletion mutants [79]. This deregulation is possibly due to indirect effects, arising from altered intracellular Ni^2+^ concentrations occurring in the *nikR* mutant strain that may interfere with the chaperone function of GroESL, which can also act as a nickel storage protein [88]. However, a search for genomic targets using a NikR consensus sequence [81] identifies two binding sites upstream of the *hspR* and *hrcA* encoding operons, suggesting that regulation of these stress response TFs may be directly subjected to NikR transcriptional control. Finally, in analogy to *Neisseria meningitidis* [89], Fur appears also to have a positive role on a subset of targets [74], including genes important for chemotaxis and motility, interactions with the host (*fibfibBP*), or redox equilibrium (*oor*). This suggests an intriguing direct link between the metal response module, virulence, and the flagellar circuit, which certainly deserves further investigation.

## Conclusion: A Shallow and Densely Interconnected TRN

The systematic analysis of origons strongly suggests that *H. pylori* adopts a shallow multilayer TRN, displaying a low level of hierarchy. The four main origons do not appear to exist as segregated regulatory modules. In fact, separate environmental inputs are interpreted by different combinations of small sets of TFs or associated proteins. For example, the flagellar FlgS histidine kinase also controls the regulation of acid response genes through ArsR. This is of broad interest, as the specificity and the biological significance of crosstalk between signal transduction pathways are vividly debated in the prokaryotic community [90], [91]. Even more striking is the metal response module, which feeds into all other origons. In particular, Fur represents a key regulator, possessing the features of a regulatory “hub” in *H. pylori*, a role played by pleiotropic regulators such as Crp, Fnr, H-NS, and Fis in *E. coli* and other bacteria [15], [92].

Another peculiar feature is the prime use of negative interactions that endow the *H. pylori* TRN to constantly monitor the environment and to quickly respond only when a regulatory signal has to be transduced. Moreover, we notice that long regulatory cascades of sequentially expressed TFs, involved in bacterial differentiation (e.g., biofilm formation and sporulation) [92], are virtually absent in this TRN. Together, the data indicate that the TRN of *H. pylori* is unambiguously built to maintain homeostasis. It is not tailored to adapt to many environmental stimuli, and apparently not flexible enough to react to metabolic signals encountered outside of the gastric niche. Possibly, the shallow network design has evolved by selecting for particular transcriptional interactions to respond to prevalent environmental inputs, in line with studies of evolutionary dynamics of bacterial TRNs, which postulate that motif tinkering allows for specific responses [14].

Several important aspects concerning the TRN remain to be elucidated. Robust discriminative binding consensus motifs are missing for many *H. pylori* TFs. Moreover, responses to oxidative and osmotic stresses have been overlooked to date, and open questions remain concerning the regulation of virulence factors and toxins promoting the inflammatory response in the host. Certain genes, including members of the *cag* pathogenicity island coding for a type IV secretion system found in hypervirulent strains, are specifically expressed upon adhesion to host cells [93]. For a comprehensive understanding of the *H. pylori* TRN, it will be imperative to identify the TFs that are involved in sensing the contact with the host cells. Similarly, the regulatory circuits driving transition form the exponential to the stationary phase [84], [85], as well as differentiation into and out of the coccoid form, which has been associated with the elusive epidemiology of *H. pylori* [94], [95], need to be identified. Poorly characterized but essential regulators, such as the atypical orphan response regulators HP1043 [96] and HP1021 [97], [98], representing the 20% of TFs not covered by the TRN model, may contribute to these circuits. The recent finding of natural antisense transcripts and putative small non-coding RNAs in *H. pylori* [99], [100] indicates that non-coding RNA regulation also may contribute to the adaptive responses of the bacterium.

In conclusion, we would like to convey the idea that the low number of transcriptional regulators, together with the considerable bulk of molecular tools and literature, set *H. pylori* as a very tractable model organism to dissect and characterize transcriptional network structures involved in virulence regulation and host–pathogen interactions. In addition, specific motifs that confer peculiar response dynamics (such as the incoherent type-2 FFL of the heat shock origon) can have interesting potential as regulatory building blocks in synthetic biology.

### Note

Recent papers have provided an integrated study of the proteome, the metabolic network, and the transcriptome of the human pathogen *Mycoplasma pneumoniae* at the systems level [101]. In particular, Güell and colleagues demonstrated that in *M. pneumoniae* complex regulatory responses are maintained despite the low number of transcriptional regulators found, via extensive use of noncoding RNAs and transcriptional units that allow complex regulation mechanisms [102]. Finally, recent work by Sharma et al. [103] in *H. pylori* discovered an unexpected wealth of antisense transcripts and small RNAs, which may act as potential regulators of *cis*- and *trans*-encoded target mRNAs. This adds to the concept that infectious agents with reduced genome sizes may be excellent model organisms in which to attempt dissection of pathogenic regulatory networks at the systems level.

## Supporting Information

Dataset S1 Binary matrix of regulatory interactions for the main *H. pylori* TFs. The zipped folder includes two Excel files, one for pre-2004 versions and one for later versions.
(0.50 MB ZIP)

Figure S1 The acid acclimation origon. The acid acclimation origon is wired in two SIMs, controlled respectively, by the phosphorylated (P∼ArsR; dark green) or the unphosphorylated (ArsR; red) form of the response regulator. The phospho-transfer event is mediated by the transmembrane histidine kinase ArsS (light green) and may be promoted in the cytoplasm by FlgS (light blue) under harsh acidic conditions. The color code of operons reflects the respective signal transduction pathway, ArsS- or FlgS-dependent (light green and light blue, respectively), or P∼independent (red). TF-DNA interactions and direct transcriptional control are depicted by black lines; arrowheads denote positive regulation; hammerheads indicate negative regulation. Ectopic regulation of Fur and NikR, feeding in from the metal homeostasis origon, is depicted.
(0.40 MB PDF)

Model S1 Zipped folder containing files for the BioTapestry Editor Java plug-in (bioTapestryEditor.jnlp) and the *H. pylori* TRN model (HpTRN_ModelS1.btp).
(0.02 MB ZIP)

Text S1 Instructions for opening and browsing the *H. pylori* TRN model with BioTapestry editor and a caption for the model.
(0.03 MB DOC)
