# Supplementary figures and images for: Built Shallow to Maintain Homeostasis and Persistent Infection: Insight into the Transcriptional Regulatory Network of the Gastric Human Pathogen Helicobacter pylori
Source: PLoS Pathog. 2010 Jun 10;6(6):e1000938. doi: 10.1371/journal.ppat.1000938 (PMC2883586; doi:10.1371/journal.ppat.1000938)

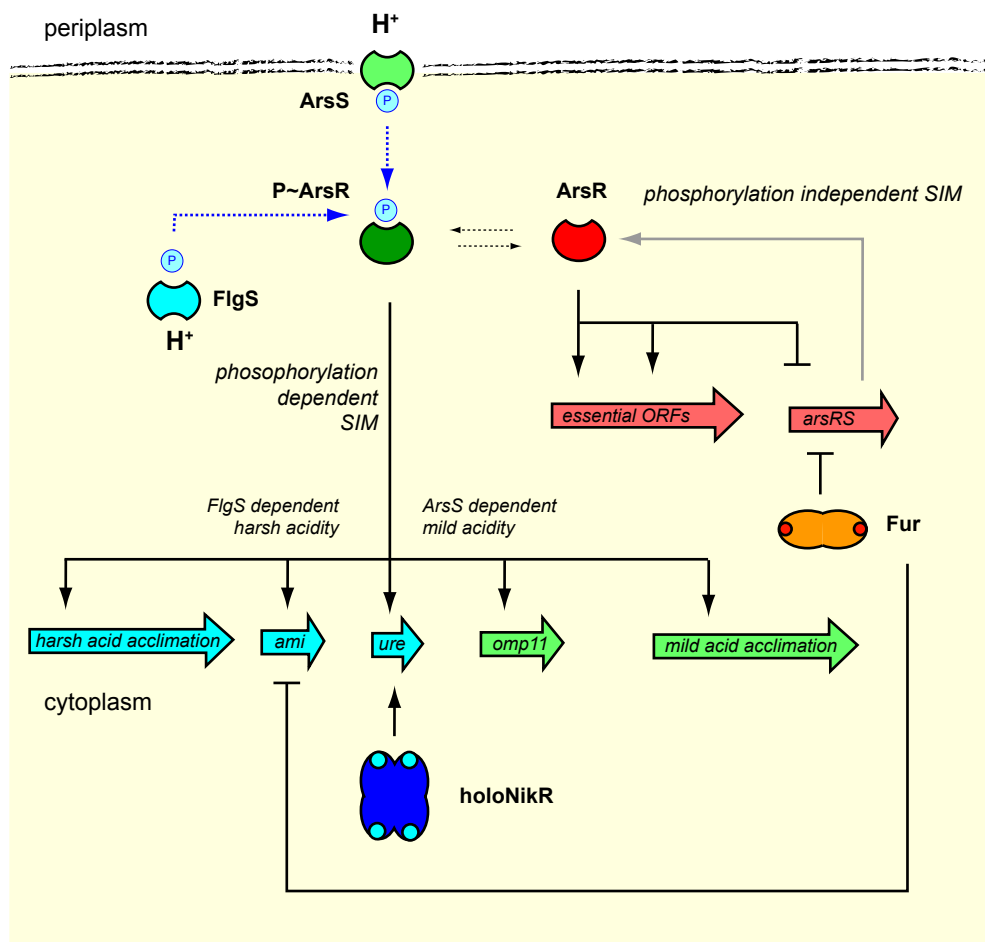

Supplement: Figure S1 — The acid acclimation origon. The acid acclimation origon is wired in two SIMs, controlled respectively, by the phosphorylated (P∼ArsR; dark green) or the unphosphorylated (ArsR; red) form of the response regulator. The phospho-transfer event is mediated by the transmembrane histidine kinase ArsS (light green) and may be promoted in the cytoplasm by FlgS (light blue) under harsh acidic conditions. The color code of operons reflects the respective signal transduction pathway, ArsS- or FlgS-dependent (light green and light blue, respectively), or P∼independent (red). TF-DNA interactions and direct transcriptional control are depicted by black lines; arrowheads denote positive regulation; hammerheads indicate negative regulation. Ectopic regulation of Fur and NikR, feeding in from the metal homeostasis origon, is depicted. (0.40 MB PDF) [file ppat.1000938.s002.pdf]
