# Supplementary material for: Built Shallow to Maintain Homeostasis and Persistent Infection: Insight into the Transcriptional Regulatory Network of the Gastric Human Pathogen Helicobacter pylori
Source: PLoS Pathog. 2010 Jun 10;6(6):e1000938. doi: 10.1371/journal.ppat.1000938 (PMC2883586; doi:10.1371/journal.ppat.1000938)
Supplement: Text S1 — Instructions for opening and browsing the H. pylori TRN model with BioTapestry editor and a caption for the model. (0.03 MB DOC) [file ppat.1000938.s004.doc]

**Built shallow to maintain homeostasis and persistent infection:**

**Insight into the Transcriptional Regulatory Network of the gastric human pathogen *Helicobacter pylori*.**

*Alberto Danielli*^1^, Gabriele Amore*^2^, Vincenzo Scarlato^1^,*

*^1^Department of Biology, via Selmi, 3, University of Bologna, 40126 Bologna, Italy*

*^2^Animal Physiology and Evolution Laboratory, Stazione Zoologica Anton Dohrn, Villa Comunale, 80121 Napoli, Italy*

# Supporting Text

### Interactive *H. pylori* TRN (Biotapestry)

Attached files: HpTRN_ModelS1.btp, bioTapestryEditor.jnlp

To open the modelled *H. pylori* TRN, launch BioTapestryEditor and open the HpTRN.btp file from the menu. If problems are encountered, solutions and updated Editors can be found and/or downloaded from the Biotapestry hompage: http://www.biotapestry.org/

### Caption for the *H. pylori* TRN (Biotapestry)

Each gene is indicated with a horizontal line (genomic DNA) topped by a bent arrow which represents the transcriptional start site (TSS). For genes encoding regulators, a line emerging from the TSS is used. Regulatory connections can be positive (arrows) negative (barred lines) or neutral (simple lines, used when the meaning of the interaction in unknown). These impinge on the DNA on the left of the TSS of target genes (in this notation representing *cis*-regulatory DNA). TF-DNA interactions are not to be considered direct unless they have been proven by *in vitro* studies; in this case a blue dot is used. Proteins are represented with ovals and a line emerging from an oval is used to represent the interaction between a specific protein and its target(s). Protein-protein interactions are represented as lines emerging from a TSS and impinging on a specific oval. For example the interaction between FlgS and FlgR is represented as a line emerging from the TSS of *flgS* impinging on the FlgR protein. Colors are used to identify the source of each line (may that be a gene or a protein). For each gene and its corresponding protein the same color is used, except in those cases (NikR and ArsR) when the same protein displays different activities upon different conditions. Colored boxes are used for environmental cues; when more than one environmental cue converge a white oval is used. Genes are represented as separated entities even if belonging to the same operon, except in the case of the *hrcA* and *cbpA* operons, where ORFs belonging to the same operon are represented as boxes downstream of a common TSS. In the TRN, three layers are recognizable: the *top layer* containing environmental cues; the *middle layer*, with transcriptional regulators and associated proteins; the *bottom layer* with effector genes. Of these latter, only some are explicitly indicated. The others are grouped inside white boxes (when data were available). Question marks are used for regulatory connections for which data are not fully consistent among different reports. Dashed lines are used when interactions are inferred but not yet formally demonstrated.

***The flagellar origon***

This representation is mostly based on the results of [32,35]. The origon is presented as a regulatory cascade with the sequential activation of *class I*, *class II*, *class III* and *intermediate class* genes. This is obtained through the activity of the *rpoD*, *rpoN* and *fliA* sigma factor encoding genes. *rpoN* is under the control of *rpoD.* However, since mutations in the *rpoN* gene do not convincingly affect the expression of *fliA*, *flhF* and *flgM* a question mark is placed on the regulatory connection between these genes and *rpoN*. According to [32], both *rpoN* and *fliA* input the *intermediate class* genes. It should be however noted that mutation on *rpoN* have a minor effect on the expression of these genes.

The RpoN protein has also a post-transcriptional positive input on FlaA [32]. A dash line connects *flgR* to the intermediate genes, because of the effect of mutations in this gene. Furthermore the activity of the HP0958 is represented according to the results of [43,46].

***The acid acclimation origon***

The diagram presented here is mostly based on the transcriptome data of [53,55-57,59,60]. Five pH conditions are considered and 5 clusters of downstream effector genes are presented: the first and the second group (group II and III of [61]) represents genes activated at pH 4.5 and 2.5 downstream of FlgS. The third [53], fourth and fifth [57] group represents genes activated at pH 5.0, 5.5 and 6.2 downstream of the activation of ArsS [60]. Signal transduction flows through ArsR for all the gene groups. However it is possible that other effectors are at work to transduce the signal from flgS (not indicated). Regulation of ArsR on *hrcA* is based on [53].

Also the connections of Fur and NikR with genes in this origon are depicted. These are based on the data of [53, 74] for Fur. On the data of [78,81,86,87] for NikR.

***The metal homeostasis origon***

In this origon all regulatory connections emanate from the Fur-NikR bifan motif. The activity of these TFs is modulated by pH and the presence of iron and nickel. The effect of pH and nickel on NikR is based on the data of [87]. The effect of iron on Fur is based on [71,81]. Binding of NikR on its own promoter has been shown in [79,81,86]. Binding of NikR on the *fur* promoter has been shown by [81]. Binding of Fur on its own promoter has been demonstrated by [89]. Binding of Fur to genes in the flagellar, acid and metal origon have been shown by [68,74]. Regulatory interactions have been shown in the same works and in [73].

Binding and regulatory interactions of NikR with downstream target genes have been demonstrated by [68,79-81].

***The heat shock origon***

The regulatory connections between HspR/HrcA and downstream target genes are based on the work of [23]. GroESL acts as chaperone for both HspR and HrcA (dotted line) and the effect of a temperature increase is the relief of the repression operated by the two TFs. A diamond is used to signify that the regulation of downstream target genes is most probably indirect and it is an output of the inter-regulation between HspR and HrcA, and GroESL titration.

The heat shock circuit receives the regulatory input from the acid and the metal origons [53,60,79]. As GroESL acts as nickel binding protein, and may modulate intracellular availability of this ion, an indirect connection with NikR is indicated.
